# Supplementary material for: SPINDOC binds PARP1 to facilitate PARylation
Source: Nat Commun. 2021 Nov 4;12:6362. doi: 10.1038/s41467-021-26588-y (PMC8568969; doi:10.1038/s41467-021-26588-y)
Supplement: Supplementary file 1 — Supplementary Information [file 41467_2021_26588_MOESM1_ESM.pdf]

SPINDOC binds PARP1 to facilitate PARylation

Supplementary Figure 1

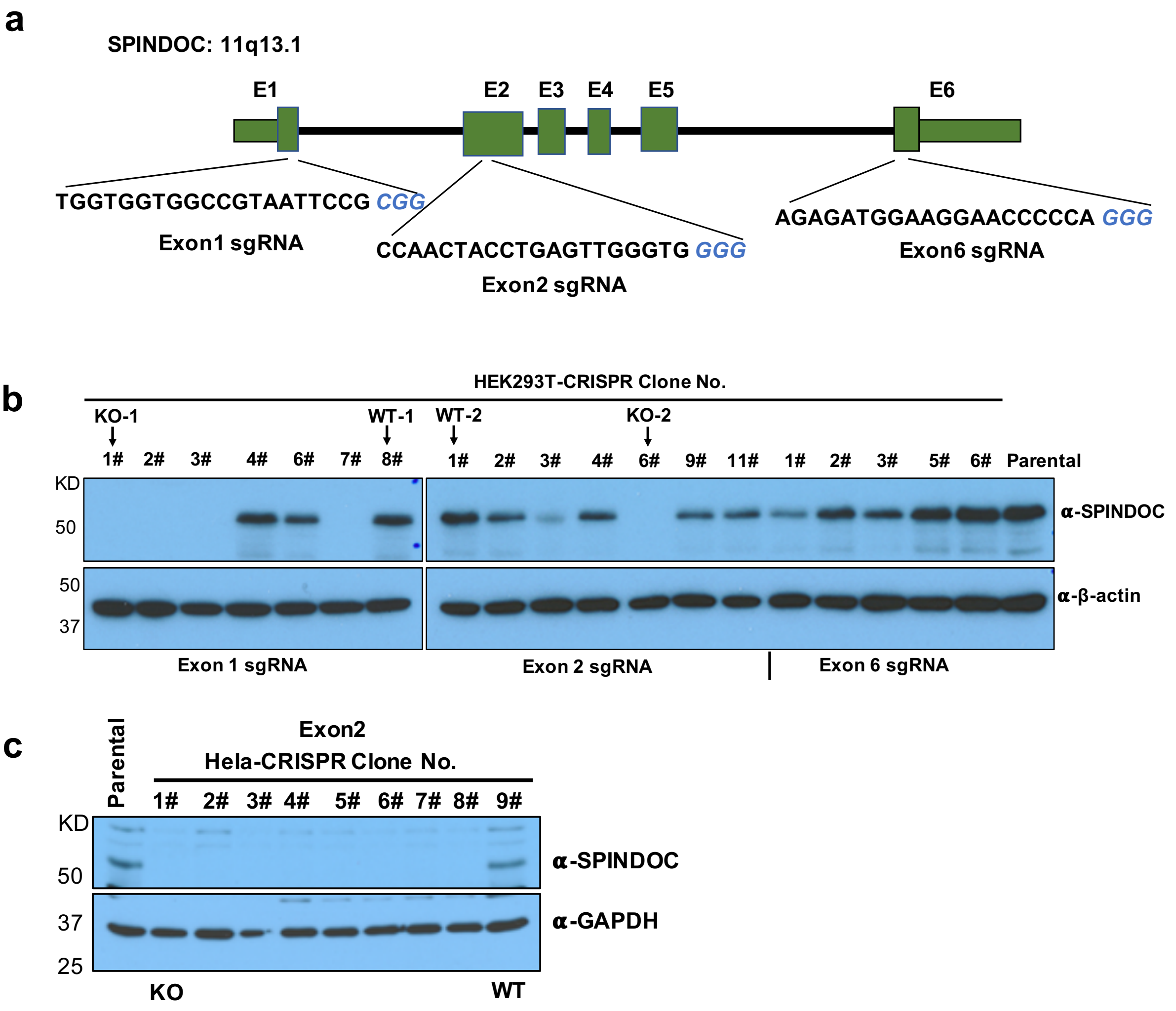

Supplementary Figure 1 Generating SPINDOC KO cell lines.

a Schematic shows the strategy of generating SPINDOC KO in human cell line. E1-E6 are exons of human SPINDOC, Exon1 sgRNA, Exon2 sgRNA and Exon6 sgRNA are shown, Pam sites are highlighted in blue. **b** Western blot shows SPINDOC KO efficiencies of single clones in HEK293T cells. For further analysis the following clones were selected: Exon1-1# as KO-1, Exon1-8# as WT-1, Exon2-1# as WT-2, Exon2-6# as KO-2. **c** Western blot shows SPINDOC KO efficiencies of single clones in Hela cells. For further analysis, Exon2-1# (Hela KO) and Exon2-9# (Hela WT) were selected.

Supplementary Figure 2

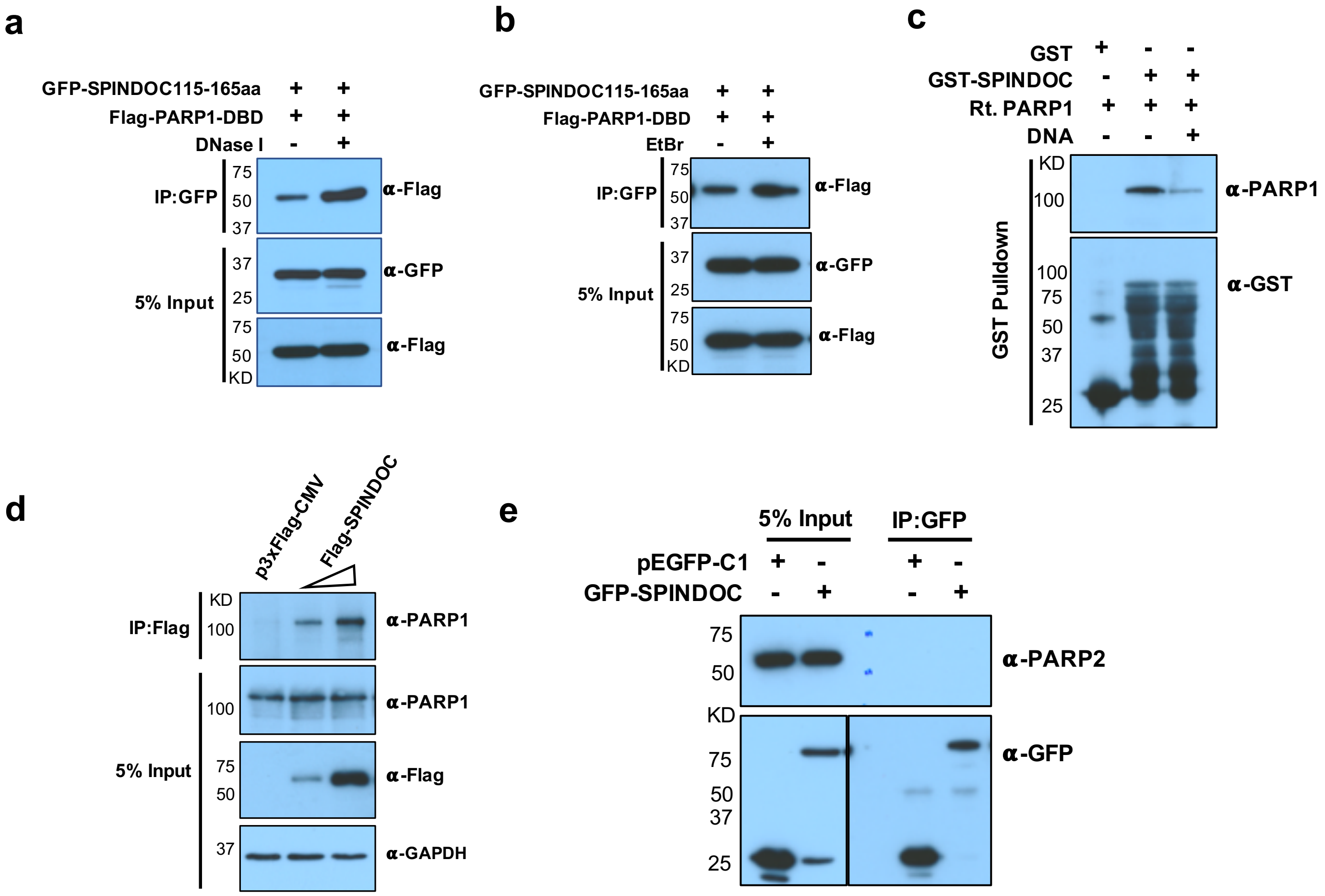

Supplementary Figure 2 GFP-SPINDOC competes with DNA for binding to PARP1.
a, b HEK293T cells were transfected with GFP-SPINDOC 115-165aa and Flag-PARP1-DBD. GFP was IPed from lysates, followed by DNase I 40U/ml treatment for 30mins on ice, and then analyzed by Western blot (**a**). GFP was IPed from lysates, followed by incubation with Ethidium Bromide (EtBr) 500 μg/ml treatment for 30mins on ice (**b**). **c** Purified GST and GST-SPINDOC proteins were co-incubated with recombinant PARP1, in the presence or absence of sheared DNA 0.2 μg/ml, then a GST pulldown assay was performed, and PARP1 was detected by Western blot analysis. **d** HEK293T cells were transfected with p3XFlag-CMV, Flag-SPINDOC 1.25μg and 5μg, and lysed before a Flag IP was performed, followed by Western blot analysis. **e** HEK293T cells were transfected with pEGFP-C1 and GFP-SPINDOC, and a GFP IP was performed to test for an interaction between PARP2 and SPINDOC.

Supplementary Figure 3

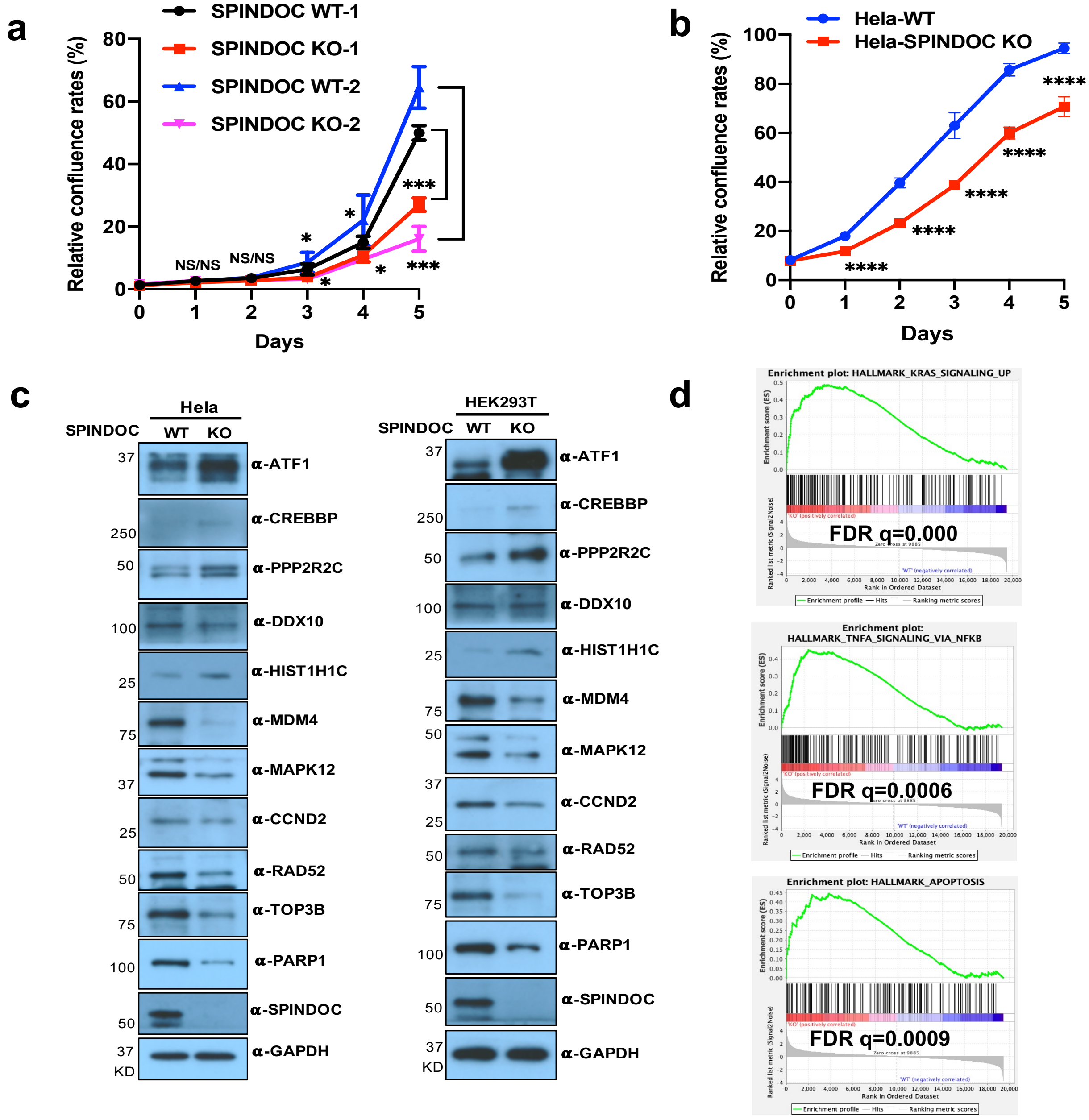

Supplementary Figure 3 SPINDOC KO cells display growth defects and altered gene expression profiles.

a HEK-293T-SPINDOC WT and KO cell lines were seeded onto 96-well plate with 1000 cells/well and cultured 5 days. Cell numbers and confluence were tested every day using a Celigo. **b** Hela-SPINDOC WT and KO cell lines were seeded onto 96-well plate with 2000 cells/well and processed as in (**a**). **c** Differentiated genes from RNA seq (**Fig. 3b**) were validated by Western blot. **d** GESA analysis of RNA seq data showed the top three hallmarks of gene set enrichments. Graphs represent mean ± SD, with * *P*<0.05, ****P*<0.001, *****P*<0.0001, NS: non-significant.

Supplementary Figure 4

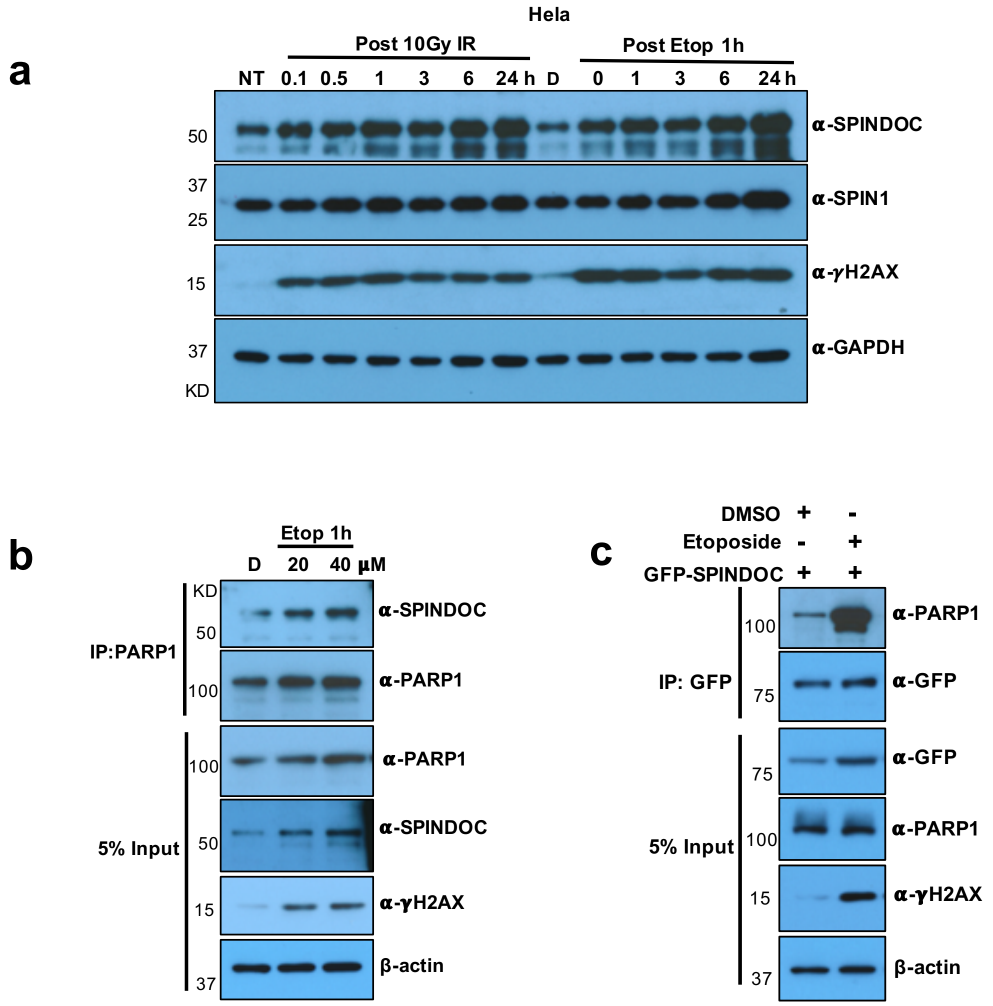

Supplementary Figure 4 DSBs induced SPINDOC expression and promotes its intera ction with PARP1.

a Hela cells were treated with 10Gy IR and 40μM Etoposide an hour for indicated timepoints and analyzed by Western blot, using the indicated antibodies. **b** HEK293T cells were treated with DMSO, Etoposide 20**μ**M and 40**μ**M an hour. Cells were then lysed and PARP1 was IPed to analyze the effect of DNA damage on the SPINDOC/PARP1interaction. **c** HEK293T cells were transfected with GFP-SPINDOC for 48 hours, followed by treatment with DMSO and Etoposide 40**μ**M an hour. Cells were then lysed and GFP was IPed to analyze the PARP1 interaction with SPINDOC.

Supplementary Figure 5

a

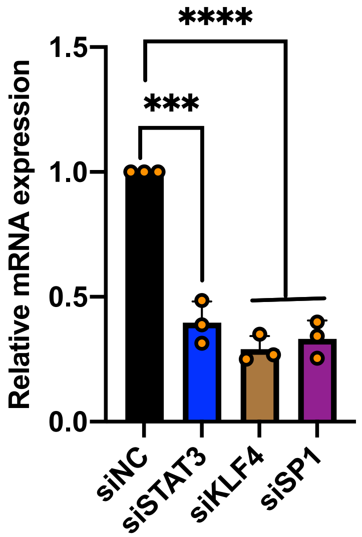

b

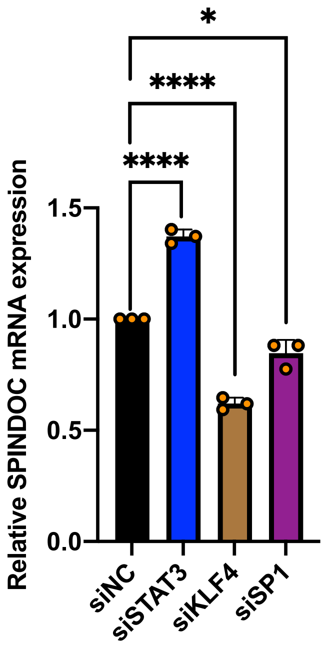

Supplementary Figure 5 Screening for transcription factor that regulate SPINDOC expression.

Hela cells were transfected with siNC, siSTAT3, siKLF4 or siSP1 for 24 hours and total RNAs were extracted for RT-qPCR. **a** Relative STAT3, KLF4 and SP1 mRNA expression showed siRNAs knockdown efficiencies. **b** Relative SPINDOC mRNA level showed effects of the loss of these 3 transcription factors on SPINDOC expression. siNC as a siRNA negative control. Graphs represent mean ± SD, with *P<0.05, ***P<0.001,****P<0.0001.

Supplementary Figure 6

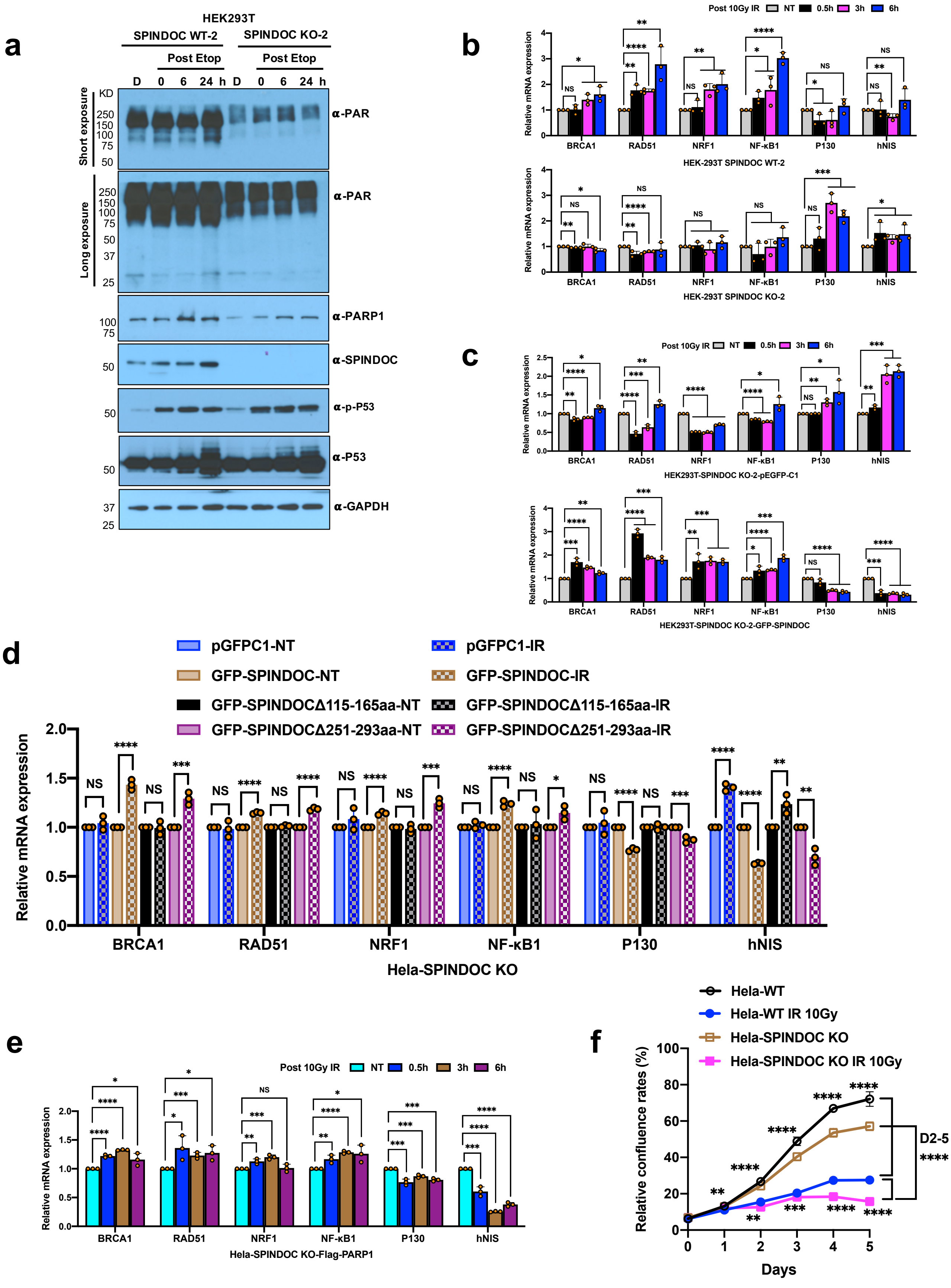

Supplementary Figure 6 SPINDOC facilitates PARylation and the transcri ption of PARP1targets.

a, HEK293T-SPINDOC WT and KO cell lines were treated with 40**μ**M Etoposide an hour, followed by 0, 6, 24 hours recovery, and then subjected to Western blot analysis. **b** HEK293T-SPINDOC WT and KO cell lines were treated with 10Gy IR, followed by 0.5, 3, and 6 hours recovery, and then subjected to RT-qPCR assay for six known PARP1 targets. **c** HEK293T-SPINDOC KO cells rescued with GFP-SPINDOC transfection were treated as the same as (**b**), pEGFP-C1 vector transfection as the control. **d** Hela-SPINDOC KO cells rescued with pEGFPC1, GFP-SPINDOC WT, △115-165aa and △251-293aa mutants were treated with 10Gy IR, followed by 0.5 hour recovery, and then subjected to RT-qPCR assay for six known PARP1 targets. **e** Hela-SPINDOC KO cells were transfected with Flag-PARP1 and then treated with 10Gy IR, followed by 0.5, 3, and 6 hours recovery, and then subjected to RT-qPCR assay for six known PARP1 targets. **f** Hela-SPINDOC WT and KO cell lines were seeded onto 96-well plate, at a density of 1000 cells/well and then subjected to IR 10Gy treatment, followed by cultured for 5 days. Relative confluency rates were obtained every day using a Celigo. D2-5 representing day 2 to day 5. Graphs represent mean ± SD, with $*P<0.05$, $**P<0.01$, $***P<0.001$, $****P<0.0001$, NS: non-significant.

Supplementary Figure 7

a

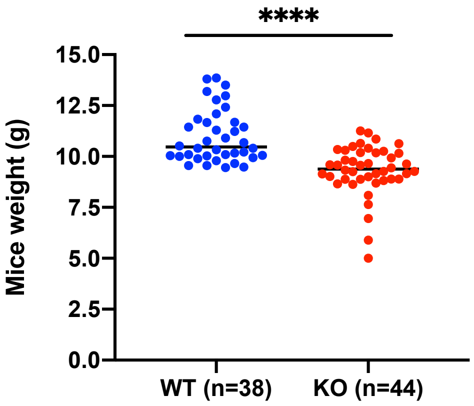

b

Genotype analysis of SPINDOC$^{+/-}$ mice offspring

Gender	WT	HE	KO
Male	30	62	31
Female	25	52	24
Total	55	114	55
Ratio	1.00	2.07	1.00

c

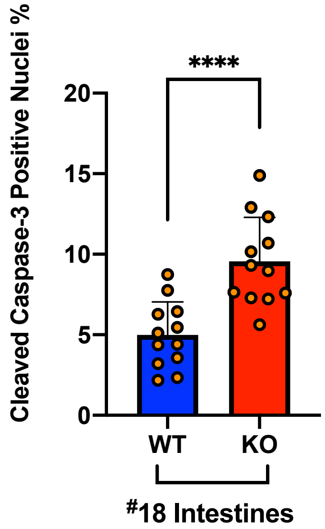

d

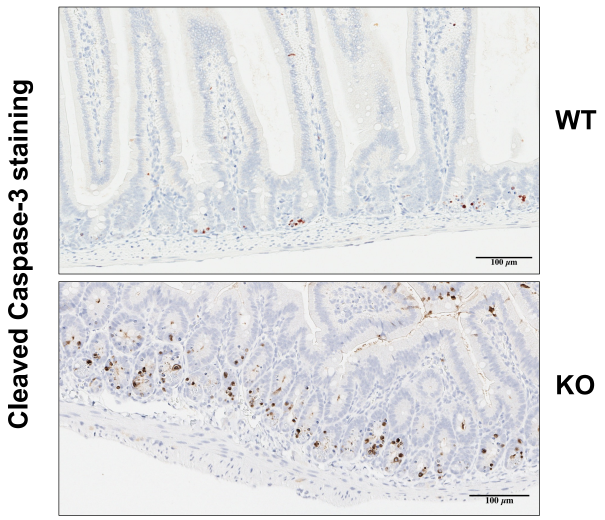

Supplementary Figure 7 SPINDOC KO mice phenotypes.

a Weights of all littermates were measured at the age of 21 days. **b** 224 pups were produced from 24 breeding pairs of SPINDOC heterozygous mice. The birth ratio of each genotype is consistent with Mendelian inheritance. **c** Small intestines taken from two pairs of line #18 SPINDOC KO/WT littermates were fixed, sectioned, and stained for cleaved Caspase-3, after 3 hours recovery from 6Gy IR treatment. Cleaved Caspase-3 positive nuclei% was shown as average value from 12 random regions (average 158331μm^2) of two slides (from two independent mice). **d** Representative IHC images of cleaved Caspase-3 stained intestine. Graphs represent mean ± SD, with ****P<0.0001.

Supplementary Table 1. Primers, sgRNA & siRNA sequences

**Human qPCR primers**		**Human qPCR primers**	
SPINDOC qPCR1-F	CAGGGAAGTGGCAGAAGGAG	CCND2-F	ATCCGCAAGCATGCTCAGAC
SPINDOC qPCR1-R	CACGCGGATAACCTGGAGAT	CCND2-R	GAGCGAGCTCACTTCCTCAT
SPINDOC qPCR2-F	GACAGCAAGGACTCACCCAA	HIST1H1C-F	GGAAGCCAAGCCCAAGGTTA
SPINDOC qPCR2-R	CACGCGGATAACCTGGAGA	HIST1H1C-R	AGCGCTCTTCTTCGGAGTTG
KLF4-F	CTGCGGCAAAACCTACACAA	MAT2A-F	CCACCCAGATAAGATTTGTGACC
KLF4-R	CGGTAGTGCCTGGTCAGTTC	MAT2A-R	GATGTAATTTCCCCAGCAAGAAG
SP1-F	CCCTTGAGCTTGTCCCTCAG	C-myc-F	CAGGACCCGCTTCTCTGAAA
SP1-R	TGAAAAGGCACCACCACCAT	C-myc-R	AACGTTGAGGGGCATCGTC
STAT3-F	CTGTGGGAAGAATCACGCCT	FASN-F	CGCGGTTTAAATAGCGTCGG
STAT3-R	ACATCCTGAAGGTGCTGCTC	FASN-R	GCAATCACCACCTCCTCCAT
NF-KB1-F	CCACCCGGCTTCAGAATGG	PARP1-F	GAAGTACGTGCAAGGGGTGT
NF-KB1-R	GCAGTGCCATCTGTGGTTGA	PARP1-R	CCAGCGGTCAATCATGCCTA
NRF1-F	GGAAAGAAAGCTGCAAGCCC	GAPDH-F	AGCCACATCGCTCAGACAC
NRF1-R	CCTGGGTCCATGAAACCCTC	GAPDH-R	GCCCAATACGACCAAATCC
hNIS-F	GTCGGCCGTGCAGGT	β-Actin-F	CTTCGCGGGCGACGA
hNIS-R	GCTGAAGCGCATCTCCAGG	β-Actin-R	CCACATAGGAATCCTTCTGACC
BRCA1-F	TCAAGGAACCTGTCTCCACA	**Mouse qPCR primers**	
BRCA1-R	TAAAGGACACTGTGAAGGCCC	mNF-KB1-F	GTCAAAATTTGCAACTATGTGGGG
RAD51-F	ATACTGTGGAGGCTGTTGCC	mNF-KB1-R	ACATGAAGTATTCCCAGGTTTGC
RAD51-R	AGTTGCAGTGGTGAAACCCA	mP130-F	CTCGCCAGCAAAGAGGAAGA
P130-F	CATGAGCGAAAGCTACACGC	mP130-R	AATGCGCACAGCAGAAGATG
P130-R	TCCTTCCACTGTCCCTTTGC	mGAPDH-F	TGTGAGGGAGATGCTCAGTG
CHEK2P2-F	CTTTATGTGGAACCCCCACCT	mGAPDH-R	TGTCCTACCCCCAATGTGT
CHEK2P2-R	GAGTCCTGTGCAGATAAAAAGAAT	**ChIP-qPCR primer**	
PPP2R2C-F	ACCTCGAAGTTTCCGAAGAAGT	SPINDOC Promoter-ChIP-F	CTTCTTAATGGAGGGAAACCCC
PPP2R2C-R	AGCTCTCCCGTGTGGTTGAA	SPINDOC Promoter-ChIP-R	CGGGTCCAGGGTTTCTAAC
MAPK-12-F	CCCTGGATGACTTCACGGAC	**Mouse genotyping primers**	
MAPK-12-R	GCTTCAGGTCCCTCAGCC	Line #18 GT-F	CCCATCCCACACAGCATCAT
ATF1-F	GCAGCCACAGTTGATTATGGAA	Line #18 GT-R	AGCTCCTCACAGCCTTCAAC
ATF1-R	CTGATAAAGATGATACCTGTTGAGC	Line #19 GT-F	CATCAGCCCTGGCATCTTCA
RAD52-F	ATAAGTAGCCGCATGGCTGG	Line #19 GT-R	GGAACTCCTGCTCCCATGAC
RAD52-R	ATCCACATTCTGCTGCGTGA	Line #21 GT-F	CCCCTTTCTGCAGTAACCCA
DDX10-F	TACTTGGAGCGGCCAAAACT	Line #21 GT-R	GCTCCTCACAGCCTTCAACA
DDX10-R	AGGCCAGTTCTCTCGTAGGT	**siRNA sequences**	
TOP3B-F	AGATTGATGCAGAGCTGGTG	KLF4 siRNA-1	AGCACUACAAUCAUGGUCAAGUUCC
TOP3B-R	TTCCTCTTGAACACGTCCAG	KLF4 siRNA-2	GGAACUUGACCAUGAUUGUAGUGCUUU
MDM4-F	TCTCCGTGAAAGACCCAAGC	STAT3 siRNA-1	AGGGCAAAGGCUUACUGAUAAACTT
MDM4-R	ACTGTGATCCTGTGCGAGAG	STAT3 siRNA-2	AAGUUUAUCAGUAAGCCUUUGCCCUGC
CREBBP-F	CCCAACCCCAAAAGAGCCA	SP1 siRNA-1	GGUGCAAACCAACAGAUUAUCACAA
CREBBP-R	TCTGGAACAAGGTTCCCACTG	SP1 siRNA-2	UUGUGAUAAUCUGUUGGUUUGCACCUG

Supplementary Table 2. List of Antibodies used in this study

Antibody	Dilution	Company	Catalog number
Rabbit polyclonal anti-C11orf84	1:500	Sigma-Aldrich	HPA040128
Mouse monoclonal anti-Flag M2	1:5000; 2μg/IP	Sigma-Aldrich	F3165
Mouse monoclonal anti-β-Actin	1:4000	Sigma-Aldrich	A2228
Mouse monoclonal anti-GFP(B2)	1:1000	Santa Cruz Biotechnology	sc-9996
Mouse monoclonal anti-P53(DO-1)	1:1000	Santa Cruz Biotechnology	sc-126
Mouse monoclonal anti-GAPDH (6C5)	1:1000	Santa Cruz Biotechnology	sc-32233
Rabbit polyclonal anti-GFP	2μg/each IP	Invitrogen	A6455
Rabbit polyclonal anti-Spindlin1	1:500	Proteintech	12105-I-AP
Rabbit monoclonal anti-PARP1 (46D11)	1:1000	Cell Signaling Technology	9532s
Rabbit monoclonal anti-Poly/Mono-ADP Ribose	1:1000	Cell Signaling Technology	83732s
Rabbit monoclonal anti-Phospho-H2A.X(ser139)	1:1000	Cell Signaling Technology	9718s
Rabbit monoclonal anti-Phospho-p53 (Ser15)	1:1000	Cell Signaling Technology	9284s
Rabbit polyclonal anti-KLF4	1:1000	GeneTex	GTX101508
Rabbit polyclonal anti-MAPK12	1:1000	Proteintech	20184-1-AP
Rabbit polyclonal anti-PPP2R2C	1:1000	Proteintech	12747-1-AP
Rabbit polyclonal anti-ATF1	1:500	Proteintech	11946-1-AP
Rabbit polyclonal anti-RAD52	1:1000	Proteintech	28045-1-AP
Rabbit polyclonal anti-DDX10	1:250	Proteintech	17857-1-AP
Rabbit polyclonal anti-MDM4	1:1000	Proteintech	17914-1-AP
Rabbit polyclonal anti-CCND2	1:1000	Cell Signaling Technology	3741T
Rabbit monoclonal anti-CREBBP	1:500	Cell Signaling Technology	7389s
Rabbit monoclonal anti-TOP3B	1:1000	Abcam	ab183520
Rabbit monoclonal anti-PARP2(JG34-56)	1:1000	Invitrogen	MA5-34728
Rabbit polyclonal anti-HIST1H1C	1:1000	Proteintech	19649-I-AP

Supplementary methods

Generation of SPINDOC CRISPR/Cas9 knockout (KO) cell lines

To produce lentivirus, HEK293T cells were grown to 50% confluency in a 10cm dish, and then transfected with 4μg of the pLentiCRISPRv.2-SPINDOCsgRNA plasmid, 4μg of psPAX2 plasmid, and 2μg of pMD2G plasmid using a PEI reagent. After 6 hours, the medium was replaced with fresh DMEM containing 10% FBS. After incubation for another 48 hours, the viral supernatant was harvested and filtered through a 0.45mm filter (Millipore). Hela and HEK293T cells were infected with viral supernatant together with 8μg/ml polybrene, and the medium was replaced with 10%FBS DMEM at 24 hours post infection. At 48 h post infection, puromycin was added to the medium at a concentration of 1.5μg/ml to select cells that harbored the sgRNAs of interest. Three days post-selection, cells were lysed for Western blot analysis of SPINDOC protein expression, to evaluate SPINDOC knockdown efficiency.

To obtain SPINDOC KO cell lines, all target cells (Hela and HEK293T) were digested with trypsin and diluted to 0.5 cell/100μl in medium, and then seeded into a 96-well plate in a volume of 100μl per well. Wells with single cells were observed and preliminarily identified under a microscope 24 hours after seeding. After 7 days, monoclonal cells were transferred to 24-well plates and then transferred to 6-well plates after 3 days. When cells were confluent, a portion of cells were used to extract genomic DNA for sequence analysis to confirm the correct targeting of genomic sequences. Another portion were used to perform Western blot for testing SPINDOC KO efficiency.

Cell viability

HEK-293T and Hela SPINDOC WT and KO cell lines were seeded on 96-well plate with 1000 or 2000 cells/well in 6 replicates and cultured for 5 days. Cell numbers and confluence were evaluated every day and relative confluence rates were calculated using a Celigo (Adherent Cell Cytometer, Nexcelom). For IR treatment, Hela SPINDOC WT and KO cell lines were

planted on 96-well plate with 1000 cells/well in 6 replicates, 12 hours later, cells were treated with 10Gy IR, and then incubated for another 12 hours, before cells were counted for the first time. Cells were subsequently counted every 24 hours, for the following 4 days.

Cleaved Caspase-3 staining and immunohistochemistry (IHC)

SPINDOC KO and WT mice were subjected to IR treatment as described and sacrificed 3 hours later. Intestine tissues were collected, mounted in cassettes, fixed in 10% neutral buffered formalin for 24–48 hours, and then moved to 70% ethanol and paraffin embedded. Tissue sections were submitted to MDACC Science Park Research Histology, Pathology & Imaging Core to perform IHC for cleaved Caspase-3. Slides stained for cleaved Caspase-3 were scanned using an Aperio ScanScope imaging platform (Leica Biosystems, IL) with 20x objective at a spatial sampling period of 0.495μm/pixel. A Genie classifier algorithm was used to quantify cleaved Caspase-3-positive and -negative nuclei. Whole-slide images were viewed and analyzed using ScanScope software.

GST pulldown

For the GST pulldown assay, we inoculated a single plasmid-containing bacterial colony pGEX-4T1 or pGEX-4T1-SPINDOC into 100 ml of fresh LB Broth with antibiotic and incubate for an hour, shaking at 37°C. IPTG (Sigma) was then added to final concentration of 0.1mM and incubated for 4 hours, shaking at 30°C. Cells were then centrifuged and the pellets resuspended in 500μl of ice-cold PBS. Samples were then sonicated at 30% amplitude using twice of pulsations (0.5s on, 0.5s off) for 20s. After centrifugation, 100μl Glutathione sepharose™ 4B (GE Healthcare, Cat# 17-0756-01) were added to the supernatant, rotated overnight at 4°C, and washed beads 3 times with ice-cold 1x PBS. Next, beads were diluted in 100μl 1x mild buffer (50mM Tris-HCL pH7.5,150mM NaCl,0.1%NP-40, 5mM EDTA, 5mM EGTA, 15mM $MgCl_2$) and aliquoted 20μl as input backup. We then aliquoted 40μl of beads and co-incubated with recombinant PARP1 5μg in 450μl 1x mild buffer, in the presence or absence of sheared salmon sperm DNA (Ambion, Cat# AM9680) 0.2μg/ml, rotated an hour at

4°C. Beads were then washed 3 times using 1x mild buffer and boiled in 1xSDS loading buffer. Finally, Western blot analysis was performed with anti-GST and anti-PARP1 antibodies.
