# Supplementary material for: SPINDOC binds PARP1 to facilitate PARylation
Source: Nat Commun. 2021 Nov 4;12:6362. doi: 10.1038/s41467-021-26588-y (PMC8568969; doi:10.1038/s41467-021-26588-y)
Supplement: Supplementary file 3 — Description of Additional Supplementary Files [file 41467_2021_26588_MOESM3_ESM.pdf]

Description of Additional Supplementary Files

These datasets include the list of proteins identified in the mass spec. interaction studies (Dataset 1-3), the list of differentially expressed gene from RNA-seq experiments (Dataset 4), the GESA genes enrichment (Dataset 5), and the list of transcription factor binding sites in the SPINDOC promoter (Dataset 6).
