# Supplementary material for: SPINDOC binds PARP1 to facilitate PARylation
Source: Nat Commun. 2021 Nov 4;12:6362. doi: 10.1038/s41467-021-26588-y (PMC8568969; doi:10.1038/s41467-021-26588-y)
Supplement: Supplementary file 5 — Reporting Summary [file 41467_2021_26588_MOESM5_ESM.pdf]

nature research

Corresponding author(s): Mark T. Bedford

Last updated by author(s): 9/03/2021

Reporting Summary

Nature Research wishes to improve the reproducibility of the work that we publish. This form provides structure for consistency and transparency in reporting. For further information on Nature Research policies, see our Editorial Policies and the Editorial Policy Checklist.

Please do not complete any field with "not applicable" or n/a. Refer to the help text for what text to use if an item is not relevant to your study. For final submission: please carefully check your responses for accuracy; you will not be able to make changes later.

Statistics

For all statistical analyses, confirm that the following items are present in the figure legend, table legend, main text, or Methods section.

n/a	Confirmed	
☐	☒	The exact sample size (*n*) for each experimental group/condition, given as a discrete number and unit of measurement
☐	☒	A statement on whether measurements were taken from distinct samples or whether the same sample was measured repeatedly
☐	☒	The statistical test(s) used AND whether they are one- or two-sided<br>*Only common tests should be described solely by name; describe more complex techniques in the Methods section.*
☒	☐	A description of all covariates tested
☐	☒	A description of any assumptions or corrections, such as tests of normality and adjustment for multiple comparisons
☐	☒	A full description of the statistical parameters including central tendency (e.g. means) or other basic estimates (e.g. regression coefficient) AND variation (e.g. standard deviation) or associated estimates of uncertainty (e.g. confidence intervals)
☐	☒	For null hypothesis testing, the test statistic (e.g. *F*, *t*, *r*) with confidence intervals, effect sizes, degrees of freedom and *P* value noted<br>*Give P values as exact values whenever suitable.*
☒	☐	For Bayesian analysis, information on the choice of priors and Markov chain Monte Carlo settings
☒	☐	For hierarchical and complex designs, identification of the appropriate level for tests and full reporting of outcomes
☒	☐	Estimates of effect sizes (e.g. Cohen's *d*, Pearson's *r*), indicating how they were calculated

Our web collection on statistics for biologists contains articles on many of the points above.

Software and code

Policy information about availability of computer code

Data collection All information of the commercial devises and websites used for data collection is given in Methods section. Specific computer code is not applicable. No software was developed by us for data collection.

Data analysis Data in this manuscript are generated using commonly available commercial software and are detailed in the corresponding methods section and figure legends.

For manuscripts utilizing custom algorithms or software that are central to the research but not yet described in published literature, software must be made available to editors and reviewers. We strongly encourage code deposition in a community repository (e.g. GitHub). See the Nature Research guidelines for submitting code & software for further information.

Data

Policy information about availability of data

All manuscripts must include a data availability statement. This statement should provide the following information, where applicable:

- Accession codes, unique identifiers, or web links for publicly available datasets
- A list of figures that have associated raw data
- A description of any restrictions on data availability

The RNA-seq GEO accession number is GSE167306. All data supporting the findings of this study are available within the article and its supplementary information files. Additional information and relevant data will be available from the corresponding author upon reasonable request. All listed figures have associated raw data.

Field-specific reporting

Please select the one below that is the best fit for your research. If you are not sure, read the appropriate sections before making your selection.

☒ Life sciences ☐ Behavioural & social sciences ☐ Ecological, evolutionary & environmental sciences

For a reference copy of the document with all sections, see nature.com/documents/nr-reporting-summary-flat.pdf

Life sciences study design

All studies must disclose on these points even when the disclosure is negative.

Sample size	Sample size was based on traditional experimental approach in molecular and cell biology. For quantitative experiments, like qPCR, samples were prepared in triplicates. The sample sizes for mice studies, 3 mice per genotype per treatment conditions were used for qPCR and 2 mice per genotype per treatment conditions were used for Western blot. For cell viability, samples were prepared in 3-6 replicates. Fluorescence intensity was calculated from 10 individual cells.
Data exclusions	No data were excluded.
Replication	The minimum 3 biological replicates were performed in each experiment in the study. For RNA seq, using two pairs of SPINDOC KO cell lines vs WT cell lines, this was 2 biological replicates. All attempts at replication were successful.
Randomization	The experiments did not require sample randomization. Samples were handled by the same way in all experiments.
Blinding	The investigators were not blinded during data collection and assessment. All approaches are considered standard for biochemical experiments performed in this study. Patient samples were not used, thus no blinding was applied.

Reporting for specific materials, systems and methods

We require information from authors about some types of materials, experimental systems and methods used in many studies. Here, indicate whether each material, system or method listed is relevant to your study. If you are not sure if a list item applies to your research, read the appropriate section before selecting a response.

Materials & experimental systems

n/a	Involved in the study	
☐	☒	Antibodies
☐	☒	Eukaryotic cell lines
☒	☐	Palaeontology and archaeology
☐	☒	Animals and other organisms
☒	☐	Human research participants
☒	☐	Clinical data
☒	☐	Dual use research of concern

Methods

n/a	Involved in the study	
☒	☐	ChIP-seq
☒	☐	Flow cytometry
☒	☐	MRI-based neuroimaging

Antibodies

Antibodies used	All antibodies used in this study were listed in Supplementary Table 2 with their source and catalog number.
Validation	Antibodies suitable for specific purposes were purchased and the validation was performed by the manufacturers. In addition, the SPIN1 and SPINDOC antibodies were validated by knock down and knockout. For more details, each antibody used in this study is provided with Research Resource Identifiers in Supplementary Table 2.

Eukaryotic cell lines

Policy information about cell lines

Cell line source(s)	HEK293T and Hela cell lines were purchased from ATCC. HEK293T-SPINDOC KO and WT cell lines were generated from HEK293T cell line. Hela-SPINDOC KO and WT cell lines were generated from Hela cell line.
Authentication	We did not authenticate these cell lines, as they were freshly puchased from ATCC.
Mycoplasma contamination	All cell lines used in this study were routinely tested for mycoplasma by using MycoAlertTM (Lonza), all negative.
Commonly misidentified lines (See ICLAC register)	No commonly misidentified lines were used in this study

Animals and other organisms

Policy information about studies involving animals; ARRIVE guidelines recommended for reporting animal research

Laboratory animals	Wild type, SPINDOC KO mice of FVB strains are used in this study. All genotypes mice were produced in house. Mice of both sex were randomly allocated to experiments at the age of 4 to 5 weeks. A detailed description of maintenance of mice is given in Methods section.
Wild animals	No wild animals were used in this study.
Field-collected samples	No field collection was performed.
Ethics oversight	All mouse experiments were reviewed and approved by the Institutional Animal Care and Use Committee at MD Anderson Cancer Center (ACUF# 00001090-RN02).

Note that full information on the approval of the study protocol must also be provided in the manuscript.

nis checklist template is licensed under a Creative Commons Attribution 4.0 International License, which permits use, sharing, adaptation, distribution and reproduction in any medium or format, as long as you give ppropriate credit to the original author(s) and the source, provide a link to the Creative Commons license, and indicate if changes were made. The images or other third party material in this article are included in e article's Creative Commons license, unless indicated otherwise in a credit line to the material. If material is not included in the article's Creative Commons license and your intended use is not permitted by atutory regulation or exceeds the permitted use, you will need to obtain permission directly from the copyright holder. To view a copy of this license, visit http://creativecommons.org/licenses/by/4.0/
